# Barriers and facilitators to healthcare access among Sub-Saharan African migrants in Europe: A scoping review

**DOI:** 10.1371/journal.pone.0351011

**Published:** 2026-06-10

**Authors:** Adanze Nge Cynthia, Jude Tsafack Zefack, Faustus Ajamah, Selouis Fuanyi Nkengfua, Emmanuel Mouladje Tchuela, Esua Alphonsius. F

**Affiliations:** 1 Sankofa Research and Mentorship Hub, Buea, Cameroon; 2 School of Global Health and Bioethics, Euclid University, Bangui, Central African Republic; 3 School of Health Sciences, Messa-Catholic University of Central Africa, Yaoundé, Cameroon; 4 Faculty of Health Science, Department of Public Health, University of Buea, Buea, Cameroon‌‌; Gulu University, UGANDA

## Abstract

**Background:**

Sub-Saharan African (SSA) migrants represent a significant and growing proportion of Europe's diverse migrant population; yet face substantial barriers in accessing healthcare services. Understanding these barriers is essential for developing equitable healthcare policies and anticipating long-term care needs as these populations age.

**Methods:**

This scoping review systematically mapped and synthesised existing evidence on healthcare access, utilization, barriers, and facilitators among SSA migrants across European settings. The primary review question was: What are the barriers and facilitators to healthcare access and utilization among Sub-Saharan African migrants in Europe? Following Arksey and O'Malley framework and PRISMA-ScR guidelines, we systematically searched Europe PMC, Scopus, Web of Science, PubMed, and Google Scholar. Fourteen studies met inclusion criteria. Data were synthesised using thematic analysis to identify descriptive and analytical themes.

**Results:**

Three overarching analytical themes emerged: (1) structural determinants as persistent barriers to care, including health insurance gaps, legal precarity, and administrative complexity; (2) intersectional vulnerabilities shaping healthcare experiences, particularly among undocumented migrants, women, and those living with HIV; and (3) cultural mediation and system misalignment, characterized by language barriers and inadequate cultural competency. Only one study specifically examined ageing and chronic care decision-making. Studies predominantly employed qualitative designs (n = 7) and cross-sectional surveys (n = 4). SSA migrants encounter multi-layered barriers that compound over time, with critical implications for health trajectories as populations age.

**Conclusion:**

Findings underscore the urgent need for migrant-sensitive healthcare approaches that address structural inequities, enhance cultural competency, and integrate long-term care planning. Future research should prioritize longitudinal studies examining healthcare needs across the life course and policy evaluations of migrant-inclusive interventions.

## Introduction

Europe hosts approximately 36% of the global migrant population, yet less than half of the member states in the World Health Organization (WHO) European Region systematically report health data for migrants, revealing a systemic failure to prioritize their wellbeing [1]. Sub-Saharan African (SSA) migrants represent a significant and growing proportion of Europe's diverse migrant population, concentrated primarily in the United Kingdom, France, Italy, Spain, and Portugal [2,3]. Despite increasing migration flows from SSA countries driven by conflict, socioeconomic instability, and climate challenges, there remains a critical paucity of research examining how these populations navigate healthcare access in European contexts [4,5]. The 2024 Lancet Series on Migration and Health Inequity in Europe emphasizes that migrants face legal, structural, linguistic, and cultural barriers that systematically exclude many from national healthcare systems, with widespread biases and inadequate cultural competency among healthcare providers’ further worsening disparities in access to preventive and clinical care [1,6]. SSA migrants encounter unique challenges shaped by recent migration patterns, distinct settlement experiences, and cultural frameworks that often conflict with European healthcare delivery models [7,8]. According to available data, SSA communities in Europe face significant obstacles to accessing healthcare, such as a lack of health insurance, language barriers, discrimination in medical settings, legal precarity, and cultural misunderstandings between patients and doctors [9–11]. A recent qualitative study in the United Kingdom (UK) found that SSA migrants experienced challenges navigating healthcare systems, fragile patient-doctor relationships, and reliance on self-medication practices when accessing healthcare [12]. Similarly, research across multiple European countries demonstrates that undocumented migrants face significant barriers including fear of deportation, financial constraints, and restricted entitlements that lead to delayed care-seeking and reliance on alternative health strategies [13,14].

The intersection of migration status, gender, socioeconomic position, and health conditions creates compound vulnerabilities that shape healthcare experiences and outcomes [15–17]. These cumulative barriers across the life course have profound implications for health trajectories as migrants age, potentially contributing to premature morbidity, unmet chronic care needs, and increased healthcare costs due to delayed interventions [18]. Understanding patterns of healthcare access and utilization across the adult life span is essential for anticipating and addressing the future long-term care needs of ageing SSA populations in Europe. Despite growing recognition of migrant health as a priority in European health policy discourse, research specifically examining SSA migrants’ healthcare experiences remains fragmented and methodologically limited [19,20].

Existing studies predominantly focus on specific subgroups such as asylum seekers, undocumented migrants, or those living with HIV, with limited comprehensive synthesis across diverse SSA populations and European contexts [21–22]. This evidence gap impedes the development of culturally responsive, equitable healthcare policies and services. Therefore, this scoping review aims to systematically map and synthesise existing evidence on healthcare access, utilization, barriers, and facilitators among SSA migrants across European settings. By examining healthcare experiences throughout the adult life course, we seek to identify structural determinants, intersectional vulnerabilities, and system misalignments that shape care-seeking behaviors and health outcomes. The specific objectives are: (1) to map the nature, scope, and geographical distribution of research on healthcare experiences among SSA migrants in Europe; (2) to identify and synthesise key barriers, facilitators, and the role of family/community networks in healthcare access and decision-making; and (3) to highlight evidence gaps and provide recommendations for developing migrant-sensitive healthcare approaches that advance health equity for this marginalized population. Through this comprehensive synthesis, we aim to provide evidence-based recommendations for developing culturally responsive long-term care approaches that effectively serve this growing and vulnerable population.

## Methods

### Study design

This scoping review was conducted to map, analyze, and synthesize existing evidence on healthcare access, utilization, and care experiences among SSA migrants living in Europe, with particular relevance to ageing trajectories and long-term care (LTC) needs. The review followed the methodological framework proposed by Arksey and O’Malley [23], further refined by Levac et al [24], and was reported in accordance with the PRISMA Extension for Scoping Reviews (PRISMA-ScR) guidelines (S1 File) [25]. In accordance with the Joanna Briggs Institute (JBI) scoping review methodology [26], the review was guided by a clearly defined primary review question: “What are the barriers and facilitators to healthcare access and utilisation among Sub-Saharan African migrants in Europe?” This question was developed to align with the study objectives and eligibility criteria, ensuring coherence between the conceptual scope, the search strategy, and the inclusion framework. No formal protocol was registered with PROSPERO or the Open Science Framework prior to the conduct of this review. This decision reflects the exploratory and mapping purpose of the review, which did not meet PROSPERO’s eligibility criteria for prospective systematic review registration.

### Eligibility criteria

#### Inclusion criteria.

Studies were included if they:

i. Focused on Sub-Saharan African migrants
ii. Were conducted in European countries
iii. Examined healthcare access, utilization, experiences, barriers, or facilitators
iv. Used qualitative, quantitative, or mixed-methods designs
v. Were peer-reviewed journal articles
vi. Were published in English

Studies that included both SSA migrants and migrants from other regions (if they reported SSA-specific findings separately, or if the majority of the study sample comprised SSA migrants and disaggregated data were extractable).

#### Exclusion criteria.

Studies were excluded if they:

i. Focused exclusively on non-African migrant groups
ii. Were conducted outside Europe
iii. Did not address healthcare access, utilization, or experiences
iv. Where SSA-specific data could not be isolated from broader migrant population findings

### Information sources and search strategy

A comprehensive electronic search was conducted between January and October 2025 across five databases: Europe PMC, Scopus, Web of Science, PubMed, and Google Scholar. No date restriction was applied to capture the full historical scope of available evidence. The search was limited to peer-reviewed articles published in English. The core Boolean search string, adapted with database-specific syntax where required, was as follows:

(“migrant” OR “migration” OR “immigrant” OR “sub-Saharan African” OR “Black African” OR “African migrant”) AND (“health care” OR “healthcare” OR “medical access” OR “health service utilization” OR “healthcare access” OR “health-seeking behavior”) AND (“Europe” OR “European”)

In Europe PMC and PubMed, MeSH terms were additionally applied, including Emigrants and Immigrants, Africa South of the Sahara, and Health Services Accessibility. In Scopus and Web of Science, searches were conducted across Title, Abstract, and Keywords fields. Google Scholar was searched using the simplified string and the first 10 pages of results were screened for relevance (S2 File). This approach is consistent with established methodological guidance recommending that Google Scholar screening be restricted to the first 10 pages (200 results) in evidence reviews, given that beyond this threshold, results exhibit rapidly diminishing relevance to the search query [27]. Notwithstanding, we acknowledge that this restriction may have resulted in missed records and should be considered a potential source of selection bias. The search yielded 112 total records (Europe PMC: n = 19; Scopus/Web of Science: n = 51; PubMed: n = 13; Google Scholar: n = 29), from which duplicates were removed, leaving 111 unique records for screening.”

### Study selection process

All retrieved records were imported into Rayyan, a web-based systematic review management tool. After removal of duplicates, 111 unique records remained for screening.

Three reviewers independently screened titles and abstracts for relevance.

**Fig 1:**
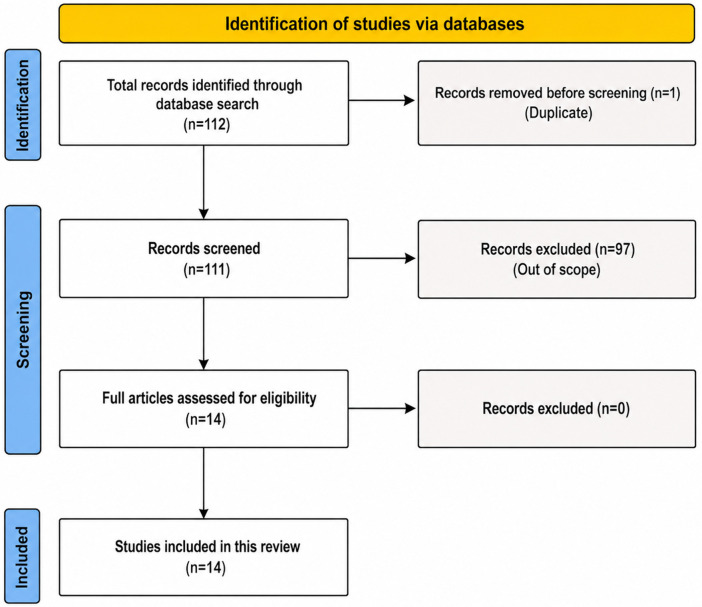
PRISMA-ScR Flow Diagram‌‌.

Any conflicts were resolved through discussion and consensus. Full-text screening was subsequently conducted independently by the same reviewers. Following full-text review, 14 studies met all inclusion criteria and were included in the final synthesis as seen in the PRISMA flow diagram in Fig 1 below.

### Data charting process

A standardized data extraction form was developed and piloted by the review team. Data were charted using Microsoft Excel to ensure transparency and auditability. The following variables were extracted from each included study:

- Author(s) and year of publication
- Country of study
- Study design and methodology
- Sample size and population characteristics
- Key findings relevant to healthcare access and experiences
- Descriptive themes
- Analytical themes

Data extraction was conducted independently by two reviewers, with discrepancies resolved through consensus or consultation with a third reviewer. When conflicts arose during extraction, the two primary reviewers first attempted resolution through structured discussion. If consensus was not reached within two review cycles, the discrepancy was escalated to a third independent reviewer (EAF or ANC), who’s judgment was final. All resolved conflicts and the rationale for adjudication decisions were documented in the extraction log to ensure auditability and methodological transparency. The data extraction form was piloted on a sample of two studies prior to full-scale extraction, and refinements were made before proceeding

### Critical appraisal of individual sources of evidence

Although formal critical appraisal is not a requisite component of scoping reviews, the methodological characteristics of included studies were informally assessed by two independent reviewers to contextualize findings; study design, sample size, and population heterogeneity were noted for each source and used to inform the interpretation of synthesized evidence rather than to exclude studies.

### Data synthesis and analysis

A thematic synthesis approach as described by Thomas and Harden (2008) was used to synthesize qualitative and mixed evidence [28]. Given that the review included quantitative studies (cross-sectional surveys and registry-based analyses), a convergent integrated approach was adopted for data synthesis, consistent with established methodology for mixed-evidence scoping reviews [29]. In this approach, quantitative findings were transformed into qualitative themes by extracting key outcomes, patterns, and statistically significant associations from each quantitative study and recasting them as descriptive statements (e.g., a registry study finding that uninsured migrants had significantly lower healthcare utilization was coded as “insurance as a structural barrier”). These transformed quantitative contributions were then integrated into the broader thematic synthesis alongside qualitative and mixed-methods findings, allowing convergent interpretive themes to emerge across all evidence types. This ensured that structural data from population-level studies meaningfully contributed to and enriched the analytical themes. This approach was selected for its suitability in integrating findings across diverse study designs and generating higher-order interpretive insights. The synthesis process unfolded through three iterative stages. Initially, line-by-line coding was conducted, whereby relevant findings from the results sections were coded inductively by two reviewers. These codes were subsequently grouped into broader descriptive themes that captured recurring patterns related to healthcare access, utilization, and lived experiences among SSA migrants. The final stage involved generating analytical themes by interpreting the descriptive themes in relation to broader theoretical perspectives, thereby enabling the synthesis to transcend simple aggregation and achieve deeper conceptual understanding. Regular team meetings were held to refine themes and ensure analytical rigor. Any disagreements were resolved through consensus, with arbitration by a third reviewer when required.

## Results

### Characteristics of included studies

The included studies used a variety of methodological techniques, including focus groups and qualitative interviews (n = 7), cross-sectional surveys (n = 4), registry-based observational studies (n = 2), and mixed-methods designs (n = 1). Sample sizes ranged from eight participants in in-depth qualitative studies to more than 1.2 million individuals in population-level registry analyses.

Included study populations comprised SSA migrants with a range of legal statuses (asylum seekers, refugees, undocumented migrants, and insured residents), varied genders, durations of stay, and health profiles. Several studies focused on particular subgroups, including women, HIV-positive individuals, asylum seekers, or undocumented migrants. Factors indirectly relevant to long-term care, including chronic illness management, long-term healthcare utilization, and structural access barriers, were evident across several studies; however, only one study explicitly examined ageing and chronic care decision-making among migrant women. Table 1 below presents a comprehensive summary of study features and major conclusions.

**Table 1:** Characteristics and Key Findings of Included Studies.

Author (Year)	Country	Study Design / Methods	Sample Size / Population	Key Findings	Descriptive Themes	Analytical Themes
Gao & Zhu 2025 [30]	Spain	Cross-sectional survey; structural equation modelling	384 SSA women	Higher host-country language proficiency reduced acculturative stress and improved access to sexual and reproductive health services	Language barriers; psychosocial stress	Cultural & social mediation
Silva et al. 2025 [31]	UK	Qualitative interviews	27 migrants from 17 countries	Difficult system navigation, self-medication practices, and fragile patient–doctor relationships shaped healthcare access and antibiotic use	System navigation; self-management	Structural determinants
Çilenti et al. 2021 [32]	Finland	Cross-sectional survey	1,795 migrants; 1,406 general population	Migrants reported higher unmet healthcare needs and lower satisfaction; reliance on municipal healthcare services	Unmet needs; service dissatisfaction	System misalignment
Isaacs et al. 2020 [33]	Scotland	Qualitative interviews	24 SSA asylum seekers	Asylum system, racism, poverty, and language barriers had sustained negative effects on physical and mental health	Structural violence; asylum policy	Structural determinants
Grupp et al. 2019 [34]	Germany	Mixed-methods (vignette-based survey and focus groups)	119 survey participants; 26 qualitative participants	Preference for religious leaders and General Practitioners (GPs); barriers to accessing psychological care	Cultural beliefs; mental health access	Cultural mediation
Müllerschön et al. 2019 [35]	Germany	Cross-sectional community-based participatory survey	1,919 SSA migrants	Lack of health insurance significantly reduced healthcare use and HIV testing; exclusion linked to shorter stay and low language proficiency	Insurance barriers; preventive care access	Structural determinants as barriers
Mbanya et al. 2019 [36]	Norway	Qualitative interviews and focus group discussions	47 SSA migrants	Barriers before and within the healthcare system included language difficulties, financial constraints, long waiting times, and dissatisfaction with providers	Structural barriers; linguistic barriers; patient experience	Structural determinants; system misalignment
Vignier et al. 2018 [37]	France	Retrospective life-event survey; mixed-effects regression	2,464 SSA migrants	Delayed health insurance acquisition; lack of residence permits and financial resources slowed access and increased risk of losing coverage	Legal and insurance barriers; gendered access; continuity of care	Structural determinants as barriers to care
Bianco et al. 2016 [38]	Italy	Cross-sectional survey	961 immigrants and refugees	High GP utilization; third-sector organizations facilitated access; preventive care remained underutilized	Role of third-sector; access facilitation	Structural facilitators
Arrey et al. 2016 [39]	Belgium	Qualitative interviews	44 HIV-positive SSA migrant women	Stigma and discrimination resulted in delayed care, emotional distress, and non-disclosure	Stigma and discrimination	Intersectional vulnerabilities
Gimeno- Feliu et al. 2015 [40]	Spain	Retrospective registry analysis	1,251,540 residents (12% immigrants)	Immigrants showed lower morbidity than natives; health deteriorated with longer duration of residence	Morbidity burden; duration of residence	Implications for ageing & LTC
Kvamme et al. 2015 [41]	Norway	Qualitative interviews	8 undocumented migrant women; 8 healthcare providers	Fear of being reported, poor language skills, and financial hardship led to delayed care and alternative health-seeking strategies	Legal precarity; gendered vulnerability; informal care	Intersectional vulnerabilities
Kristiansen et al. 2015 [42]	Denmark	Qualitative interviews (phenomenological approach)	29 migrant women (14 interviews)	Chronic illness, healthcare quality, social services, and family ties shaped decisions to age in Denmark	Ageing; chronic care needs; belonging	Implications for ageing & LTC
Diaz et al. 2015 [43]	Norway	Registry-based study	36,366 African immigrants	Primary healthcare use varied by country of origin and length of stay, with lower utilization among some SSA groups	Healthcare utilization patterns; duration of stay	Structural determinants

### Critical appraisal within sources

Across the 14 included studies, methodological quality varied by design: qualitative studies (n = 7) relied on small purposive samples limiting transferability; cross-sectional surveys (n = 4) were susceptible to selection and recall bias; registry-based studies (n = 2) offered population-level breadth but lacked individual-level contextual data; and the single mixed-methods study provided limited integration of qualitative and quantitative strands. These methodological considerations were taken into account when interpreting and weighting evidence during thematic synthesis.

### Thematic synthesis

Using thematic synthesis, three overarching analytical themes were generated from the data: 1. Structural determinants as persistent barriers to care; 2. Intersectional vulnerabilities shaping healthcare experiences, and 3. Cultural mediation and system misalignment.

These themes were derived from a set of interrelated descriptive themes, as outlined below.

1. Structural Determinants as Persistent Barriers to Care

Across the included literature, structural determinants consistently emerged as the most influential drivers of healthcare access and utilization among SSA migrants in Europe. Multiple quantitative and qualitative studies demonstrated that legal status, health insurance coverage, and administrative complexity functioned as upstream barriers that constrained timely and continuous engagement with healthcare services. In Germany, lack of health insurance was strongly associated with reduced healthcare utilization and lower uptake of HIV testing among SSA migrants, particularly among those with shorter durations of stay and limited language proficiency [35]. Similarly, life-course analyses from France revealed that delayed acquisition of residence permits and insurance coverage significantly disrupted continuity of care and increased the risk of losing coverage altogether, with pronounced gendered effects [37]. Registry-based studies further highlighted how structural exclusion translated into differential patterns of primary healthcare use over time. In Norway, Diaz et al. [43] found that healthcare utilization varied substantially by country of origin and length of residence, with persistently lower use among some SSA groups. Qualitative evidence from Scotland and Norway reinforced these findings, illustrating how asylum policies, bureaucratic complexity, and financial constraints produced sustained physical and psychological distress and discouraged preventive care engagement [33,36]. While some facilitative roles of third-sector organizations were observed in Italy, particularly in supporting general practitioner access, preventive services remained underutilized, underscoring the limits of downstream interventions in the absence of structural reform [38].

These structural obstacles were cumulative rather than episodic, undermining continuity of care and increasing the risk of unmet health needs. These trends have significant implications for aging populations, who are more likely to need ongoing, coordinated care.

2. Intersectional Vulnerabilities and Lived Experiences of Care

Beyond structural exclusion, several studies demonstrated how intersecting social positions, including gender, migration status, health condition, and socioeconomic vulnerabilities, shaped lived experiences of healthcare access. Qualitative studies among undocumented women in Norway revealed that fear of deportation, financial hardship, and limited language proficiency led to delayed care-seeking and reliance on informal or alternative health strategies, particularly for reproductive and chronic health needs [41]. Similar dynamics were observed among asylum seekers in Scotland, where structural violence embedded within immigration and asylum systems intersected with racism and poverty to produce enduring health deterioration and feelings of powerlessness [33].

Stigma and discrimination emerged as particularly salient among SSA migrant women living with HIV. In Belgium, Arrey et al. [39] documented how discriminatory encounters within healthcare settings resulted in delayed engagement, emotional distress, and selective disclosure of health status. These intersectional vulnerabilities were further compounded by gendered caregiving roles and insecure legal status, as evidenced in the French PARCOURS study, where women experienced longer delays in accessing insurance and care continuity [37]. The intersection of these vulnerabilities, worsened with time, particularly for those with chronic disease or prolonged vulnerability, with potential long-term impacts on wellbeing and care trajectories as migrants age.

Collectively, these studies illustrated how intersecting vulnerabilities accumulate across the life course, increasing the risk of unmet healthcare needs and adverse outcomes as migrant’s age.

3. Cultural Mediation and System Misalignment

Cultural mediation and misalignment between SSA migrants’ health beliefs and European healthcare systems were consistently identified as critical determinants of healthcare engagement. Language barriers were central to this misalignment, shaping communication with providers, system navigation, and perceived quality of care. In Spain, higher host-country language proficiency among SSA women was associated with reduced acculturative stress and improved access to sexual and reproductive health services, highlighting language acquisition as a protective factor [30]. Conversely, limited language skills contributed to miscommunication, dissatisfaction, and disengagement in multiple contexts, including Norway and Finland [32,36].

Cultural beliefs regarding illness, healing, and help-seeking further influenced patterns of care utilization. Among SSA asylum seekers in Germany, strong reliance on religious leaders and general practitioners for mental health concerns was observed, alongside limited engagement with specialized psychological services [34]. In the UK, migrants reported fragile patient–provider relationships and widespread reliance on self-medication and informal knowledge networks when navigating complex healthcare systems [31]. These findings collectively point to systemic shortcomings in cultural responsiveness and underscore the need for culturally mediated care pathways that bridge institutional practices and migrant lived realities.

**Fig 2:**
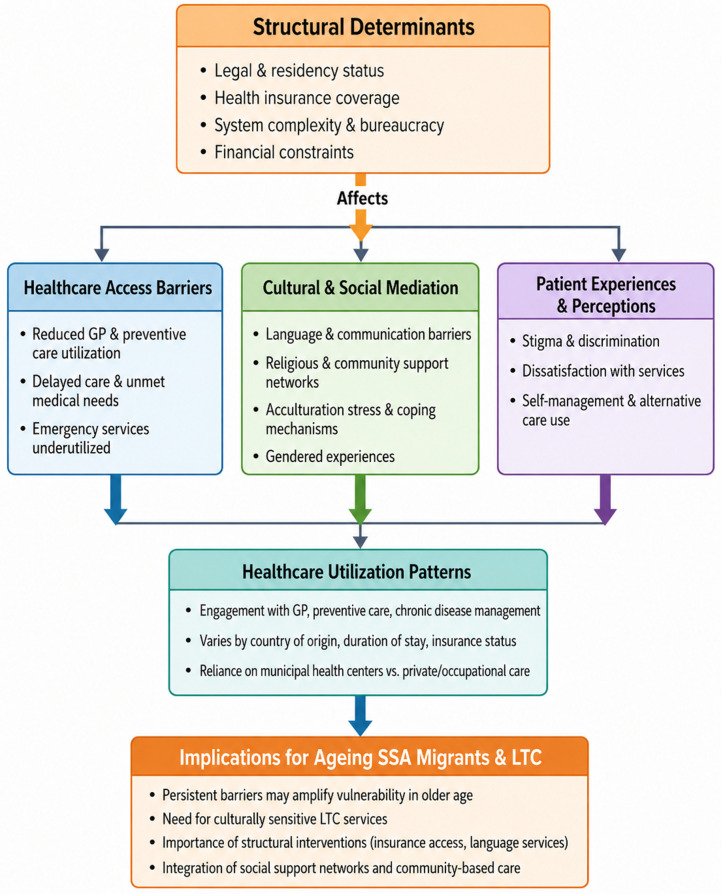
Conceptual Framework: Healthcare Experiences and LTC Implications for SSA Migrants in Europe.

The Fig 2 below presents a conceptual framework integrating our review findings with broader theoretical perspectives on migrant healthcare experiences and long-term care implications. The framework illustrates how structural determinants (legal status, insurance barriers, policy environments), intersectional vulnerabilities (gender, migration status, socioeconomic position, health conditions), and cultural/linguistic factors converge to shape healthcare access, utilization patterns, and care-seeking behaviors among SSA migrants. These proximal and distal determinants operate within the broader context of European healthcare systems and migration policies, ultimately influencing health outcomes and long-term care needs as migrant’s age. The framework emphasizes bidirectional pathways, showing how poor healthcare access and unmet health needs during working-age years cascade into increased chronic disease burden, functional limitations, and complex long-term care requirements in later life. This conceptual model can guide future research examining healthcare trajectories across the migrant life course and inform the development of comprehensive, equity-oriented interventions that address multiple levels of the healthcare ecosystem.

## Discussion

This scoping review synthesised evidence from 14 studies examining healthcare access and experiences among SSA migrants in Europe, revealing persistent structural, intersectional, and cultural barriers that systematically exclude these populations from equitable care. The predominance of structural determinants as barriers aligns with the 2024 Lancet Series on Migration and Health Inequity in Europe, which emphasises that legal frameworks, insurance systems, and administrative complexities create insurmountable obstacles for migrants, particularly those with precarious legal status [1,6].

This scoping review demonstrates that healthcare access among SSA migrants in Europe is shaped by interlocking structural, social, and cultural determinants that persist across the life course. Consistent with prior migrant health syntheses, legal status and health insurance coverage emerged as foundational determinants of access, influencing not only initial entry into healthcare systems but also continuity of care over time. Studies from Germany, France, Norway, and Spain consistently showed that lack of insurance or delayed coverage significantly reduced preventive service use, HIV testing, and chronic disease management, particularly among recent and undocumented migrants [35,37,41,43].

These findings resonate with recent evidence showing that immigrant-friendly health policies are significantly associated with better health outcomes and reduced unmet healthcare needs [44]. The cumulative, rather than episodic, nature of these structural barriers is particularly concerning for long-term care planning, as migrants with chronic conditions face compounding difficulties in accessing continuous, coordinated care across healthcare systems [45].

Intersectional vulnerabilities further mediated healthcare experiences. Qualitative evidence revealed that gender, migration status, and stigmatized health conditions, especially HIV, interacted to produce delayed care-seeking, emotional distress, and selective disclosure within healthcare encounters [33,39,41]. These findings align with contemporary research demonstrating how racism, xenophobia, and discriminatory policies in European health systems exacerbate health inequities and violate international human rights obligations [46]. The intersection of these vulnerabilities has profound implications for ageing trajectories, as migrants with prolonged exposure to structural violence and inadequate chronic disease management are at heightened risk for premature morbidity and accelerated health deterioration [17,47].

Cultural mediation and system misalignment were pervasive across settings. Language barriers undermined patient–provider communication, system navigation, and satisfaction, while cultural beliefs influenced preferences for general practitioners, religious leaders, and self-management strategies [30,34,36]. Importantly, improved host-country language proficiency was associated with reduced acculturative stress and improved access to sexual and reproductive health services, highlighting language support as a modifiable intervention point [30].

These findings align with recent systematic evidence demonstrating that approximately 30% of migrants across Europe report language problems as primary barriers to healthcare access [10]. The cultural mismatch between European healthcare delivery models and SSA cultural frameworks, particularly regarding mental health care, where participants expressed preferences for religious leaders and general practitioners over professional psychological services [34], reflects inadequate cultural competency training among healthcare providers. Recent WHO European Region assessments confirm the absence of consistent, standardized intercultural competence training across European healthcare systems, contributing to culturally insensitive care delivery [9,20]. The reliance on informal translators and inadequate professional interpretation services not only compromises care quality but also raises concerns about confidentiality and accuracy in clinical communication, particularly for sensitive health issues [48].

Notably, despite widespread discussion of chronic illness and prolonged system exposure, only one study explicitly examined ageing and long-term care decision-making among SSA migrants [42]. This gap suggests that existing healthcare inequities may translate into disproportionate long-term care needs as SSA migrant populations age, reinforcing calls for migrant-sensitive, life-course–oriented health system planning.

## Limitations

Several limitations should be acknowledged. First, the included studies were heterogeneous in design, populations, and outcomes, limiting direct comparability and precluding meta-analysis. Second, most studies were cross-sectional or qualitative, restricting causal inference and longitudinal insight into healthcare trajectories over time. Third, evidence on ageing and long-term care was sparse, with limited focus on older SSA migrants despite clear relevance to chronic disease and service continuity. Fourth, the restriction of the search to English-language publications constitutes a potential language bias: studies published in French, Dutch, German, Italian, Spanish, and other European languages were not retrieved, which may have led to the exclusion of relevant evidence from non-Anglophone European settings where SSA migrant communities are well-established (e.g., France, Portugal, Italy). This should be considered an inherent limitation of the evidence base. Fifth, no formal protocol was pre-registered for this review (e.g., on PROSPERO or the Open Science Framework), which limits the transparency and replicability of the review process.

## Conclusion

This scoping review demonstrates that healthcare access for Sub-Saharan African (SSA) migrants in Europe is hindered by persistent structural barriers, cultural misalignment, and intersectional vulnerabilities that compound as these populations age. Achieving health equity requires a shift from short-term access toward systemic reforms, including universal health coverage regardless of immigration status, the integration of culturally responsive care, and the use of disaggregated data to inform long-term care planning. Future research should prioritize longitudinal studies and comparative policy evaluations co-designed with SSA communities to ensure that interventions and research agendas remain grounded in the lived experiences and evolving needs of the migrant life course.

## Supporting information

S1 File Search Strategy. (DOCX)

S2 File PRISMA-ScR-Checklist. (DOCX)
