# Supplementary material for: Barriers and facilitators to healthcare access among Sub-Saharan African migrants in Europe: A scoping review
Source: PLoS One. 2026 Jun 10;21(6):e0351011. doi: 10.1371/journal.pone.0351011 (PMC13252806; doi:10.1371/journal.pone.0351011)
Supplement: S1 File — (DOCX) [file pone.0351011.s001.docx]

**Supplementary File 1**

**Search Strategies**

**Information Sources**

**A comprehensive database search was conducted across the following electronic databases:**

- Europe PMC (n = 19 records)
- Scopus and Web of Science (n = 51 records)
- PubMed (n = 13 records)
- Google Scholar (n = 29 records)

**Search Strategies by Database**

**1. PubMed Search Strategy**

Total records retrieved: 13

The following Boolean search string was applied in PubMed:

("migrant" OR "migration" OR "sub-Saharan" OR "Black African")

AND

("health care" OR "medical access")

*Database-specific MeSH adaptations were applied where applicable. The search was conducted without date restrictions. All retrieved records were exported for deduplication and screening.*

**2. Scopus and Web of Science Search Strategy**

Total records retrieved: 51

The following Boolean search string was applied in Scopus and Web of Science:

("migrant" OR "migration" OR "sub-Saharan" OR "Black African")

AND

("health care" OR "medical access")

*Field tags were adapted to suit each database's syntax. Searches were conducted in title, abstract, and keyword fields.*

**3. Europe PMC Search Strategy**

Total records retrieved: 19

The following Boolean search string was applied in Europe PMC:

("migrant" OR "migration" OR "sub-Saharan" OR "Black African")

AND

("health care" OR "medical access")

*Europe PMC-specific filters were applied to limit results to peer-reviewed journal articles published in English.*

**4. Google Scholar Search Strategy**

Total records retrieved: 29

The following Boolean search string was applied in Google Scholar:

("migrant" OR "migration" OR "sub-Saharan" OR "Black African")

AND

("health care" OR "medical access")

*The first ten pages of results were screened to capture the most relevant records. Since Google Scholar does not support MeSH terms; searches were conducted in full-text mode.*

Table 1. Summary number of records retrieved per database and the total after deduplication.

| **Database** | **Records Retrieved** | **Notes** |
| --- | --- | --- |
| Europe PMC | 19 | MeSH adaptations applied |
| Scopus and Web of Science | 51 | Field tags adapted |
| PubMed | 13 | MeSH terms used |
| Google Scholar | 29 | First 10 pages screened |
| **Total (before deduplication)** | **112** | **—** |
| **After deduplication (unique records)** | **111** | **Screened in Rayyan** |
