# Supplementary material for: Barriers and facilitators to healthcare access among Sub-Saharan African migrants in Europe: A scoping review
Source: PLoS One. 2026 Jun 10;21(6):e0351011. doi: 10.1371/journal.pone.0351011 (PMC13252806; doi:10.1371/journal.pone.0351011)
Supplement: Attachment — Submitted filename: Response to Reviewers.pdf [file pone.0351011.s003.pdf]

Point-by-Point Response to Reviewers

We sincerely thank the reviewer for their thorough, constructive, and expert evaluation of our manuscript. The feedback has substantially improved the methodological rigour, clarity, and transparency of the review. Below, we address each comment point by point. All changes in the revised manuscript are highlighted in red to facilitate tracking. New references added in response to reviewers' comments are numbered 26, 27, and 29 and are highlighted in red in both the text and the reference list.

REVIEWER #1, MAJOR COMMENTS

Major Comment 1:

Conceptual and Methodological Inconsistency. Reviewer comment: "The manuscript is described as a 'narrative scoping review', which is methodologically inconsistent. A review should be clearly defined as either a narrative review, or a scoping review with systematic methodology. This issue appears throughout the manuscript (including title and abstract)."

Authors' response:

We fully accept this critique. The term 'narrative scoping review' was internally contradictory and has been removed throughout the manuscript. The review is now consistently described as a 'scoping review' in the:

• Title (changed from "A Narrative Scoping Review" to "A Scoping Review")

• Abstract Methods section

• Introduction (objectives paragraph)

• Methods, Study Design section

The revised Methods section now clearly situates the review as a scoping review following the Arksey and O'Malley framework, Levac et al. refinements, and the Joanna Briggs Institute (JBI) scoping review methodology.

Major Comment 2:

Absence of an Explicit Review Question. Reviewer comment: "Although the authors state that they follow Arksey and O'Malley's framework, a clear review question is not explicitly presented, which is a fundamental requirement of scoping reviews. The authors should clearly articulate the primary review question, and demonstrate its alignment with the study objectives and eligibility criteria."

Authors' response:

We agree that the absence of an explicit review question was a significant methodological gap. We have now added a clearly stated primary review question in both the Abstract and the Methods Study Design section, as follows:

"What are the barriers and facilitators to healthcare access and utilization among Sub-Saharan African migrants in Europe?"

The alignment between this review question, the study objectives, and the eligibility criteria is now explicitly stated in the revised Methods section. The inclusion criteria (SSA migrants; European setting; healthcare access/utilization focus; any study design; peer-reviewed) directly operationalize the boundaries implied by the review question. The three specific objectives map onto the scope defined by the question.

Major Comment 3:

Use of Outdated Methodological Framework. Reviewer comment: "The study relies primarily on Arksey and O'Malley (2005), with reference to Levac et al. While foundational, this approach is now considered outdated if used alone. The manuscript would be strengthened by aligning with Joanna Briggs Institute (JBI) scoping review methodology."

Authors' response:

We thank the reviewer for this important methodological feedback. We have added explicit reference to and alignment with the JBI scoping review methodology (Peters et al., 2020) [Reference 26], which represents the most current and comprehensive guidance for scoping review conduct. The revised Methods, Study Design section now reads as follows;

"In accordance with the Joanna Briggs Institute (JBI) scoping review methodology, the review was guided by a clearly defined primary review question... The review is explicitly defined as a scoping review, not a narrative review, in keeping with the systematic and iterative methodology employed throughout."

New reference added: Peters MDJ, Godfrey C, McInerney P, Munn Z, Tricco AC, Khalil H. Chapter 11: Scoping reviews (2020 version). In: Aromataris E, Munn Z, editors. JBI Manual for Evidence Synthesis. JBI; 2020. [Reference 26]

Major Comment 4:

Quantitative Data Integration in Thematic Synthesis. Reviewer comment: "The authors report using thematic synthesis for qualitative and mixed-methods evidence. However, the review includes quantitative studies (e.g., cross-sectional surveys and registry data). It is unclear how quantitative data were incorporated into the synthesis and whether a convergent or segregated approach was used."

Authors' response:

This is a valid and important methodological concern. We have substantially revised the Data Synthesis and Analysis section to clarify how quantitative data were incorporated. Specifically, we adopted a convergent integrated approach, consistent with Hong et al. (2017) [Reference 29]. In this approach:

• Quantitative findings were transformed into qualitative representations by extracting key outcomes, patterns, and statistically significant associations and recasting them as descriptive statements (e.g., registry evidence of lower utilisation among uninsured migrants was coded as "insurance as a structural barrier").

• These transformed quantitative contributions were then integrated alongside qualitative and mixed-methods findings into the thematic synthesis, enabling convergent interpretive themes to emerge across evidence types.

New reference added: Hong QN, Pluye P, Bujold M, Wassef M. Convergent and sequential synthesis designs: implications for conducting and reporting systematic reviews of qualitative and quantitative evidence. Syst Rev. 2017; 6(1):6. [Reference 29]

Major Comment 5:

Handling of Mixed Migrant Population Studies. Reviewer comment: "The inclusion criteria specify SSA migrants, but it is unclear how studies with mixed migrant populations were handled. The authors should clarify whether such studies were included and how SSA-specific findings were extracted or interpreted."

Authors' response:

We agree that clarification was necessary. We have added a dedicated paragraph following the Inclusion Criteria in the revised manuscript that reads as follows:

"Regarding studies with mixed migrant populations: studies that included both SSA migrants and migrants from other regions were included if they reported SSA-specific findings separately, or if the majority of the study sample comprised SSA migrants and disaggregated data were extractable. Where SSA-specific data could not be isolated from broader migrant population findings, the study was excluded to preserve analytical precision."

This clarification ensures transparency regarding how population specificity was maintained throughout the review.

Major Comment 6:

Justification for Google Scholar Search Restriction to First 10 Pages. Reviewer comment: "The search strategy restricted Google Scholar screening to the first 10 pages. This approach requires justification and supporting methodological references. Otherwise, it may introduce selection bias."

Authors' response:

We appreciate this comment. We have added a methodological justification and supporting reference for this decision in the revised Information Sources and Search Strategy section.

"This approach is consistent with established methodological guidance recommending that Google Scholar screening be restricted to the first 10 pages (200 results) in evidence reviews, given that beyond this threshold, results exhibit rapidly diminishing relevance to the search query. Notwithstanding, we acknowledge that this restriction may have resulted in missed records and should be considered a potential source of selection bias."

New reference added: Haddaway NR, Collins AM, Coughlin D, Kirk S. The role of Google Scholar in evidence reviews and its applicability to grey literature searching. PLoS One. 2015;10(9):e0138237. [Reference 27]

Major Comment 7:

Protocol Registration. Reviewer comment: "There is no mention of protocol registration (e.g., PROSPERO, OSF). Indicate whether a protocol exists, or justify its absence."

Authors' response:

We acknowledge this important point. We have added a clear statement on protocol registration in both the Methods, Study Design section and the Limitations section. The revised Methods text states:

"No formal protocol was registered with PROSPERO or the Open Science Framework prior to the conduct of this review. This decision reflects the exploratory and mapping purpose of the review, which did not meet PROSPERO's eligibility criteria for prospective systematic review registration. We acknowledge this as a methodological limitation and recommend that future scoping reviews on this topic consider prospective protocol registration to enhance transparency and reproducibility."

Additionally, the Limitations section now includes: "Fifth, no formal protocol was pre-registered for this review (e.g., on PROSPERO or the Open Science Framework), which limits the transparency and replicability of the review process. Future reviews on this topic are encouraged to register their protocol prospectively."

REVIEWER #1, MINOR COMMENTS

Minor Comment 1:

Language Editing and Introduction Structure. Reviewer comment: "The manuscript requires substantial language editing. Issues include grammatical errors, incorrect article usage, and long, complex sentences. The Introduction is presented as a single extended paragraph, which reduces clarity and readability."

Authors' response:

We have undertaken careful language revision throughout the manuscript. Specifically:

• The Introduction has been restructured from a single extended paragraph into four logically organized paragraphs:

(1) Migration context and SSA population profile in Europe (refs. 1–5)

(2) Structural, legal, and cultural barriers to healthcare (refs. 6–14)

(3) Intersectional vulnerabilities and life-course implications (refs. 15–20)

(4) Research gap and review objectives (refs. 21–22)

• Grammatical errors, incorrect article usage, and overly complex sentence constructions have been corrected throughout the manuscript.

• Transitions between ideas in the Methods and Results sections have been made more concise and coherent.

Minor Comment 2:

Detail on Data Extraction and Conflict Resolution. Reviewer comment: "Providing more detail on data extraction procedures and how to solve conflicts between reviewers."

Authors' response:

We have expanded the Data Charting Process subsection to provide more detail on both the data extraction procedures and the conflict resolution process. The revised text now clarifies that:

• The data extraction form was piloted on two studies before full-scale extraction, with refinements made as needed.

• When conflicts arose, primary reviewers attempted resolution through structured discussion; if unresolved after two cycles, a third independent reviewer (EAF or ANC) made the final adjudication.

• All resolved conflicts and the rationale for adjudication decisions were documented in the extraction log to ensure auditability.

Minor Comment 3:

English-Language Restriction as an Explicit Limitation. Reviewer comment: "The restriction to English-language studies only should be explicitly acknowledged as a limitation due to potential language bias."

Authors' response:

We agree. Although the English-language restriction was mentioned in the original Limitations section, it was insufficiently elaborated. We have strengthened this limitation statement in the revised manuscript as follows:

"Fourth, the restriction of the search to English-language publications constitutes a potential language bias: studies published in French, Dutch, German, Italian, Spanish, and other European languages were not retrieved, which may have led to the exclusion of relevant evidence from non-Anglophone European settings where SSA migrant communities are well-established (e.g., France, Portugal, Italy). This should be considered an inherent limitation of the evidence base."

We believe these revisions have substantially strengthened the manuscript. We are grateful for the reviewer's expert input and remain available to address any further concerns.
