# Supplementary material for: Barriers and facilitators to healthcare access among Sub-Saharan African migrants in Europe: A scoping review
Source: PLoS One. 2026 Jun 10;21(6):e0351011. doi: 10.1371/journal.pone.0351011 (PMC13252806; doi:10.1371/journal.pone.0351011)
Supplement: Peer review history

# Peer review: Decision Letter 0

**DOI:** 10.1371/journal.pone.0351011.r001

**Authors:** Felix Bongomin

6 May 2026

PONE-D-26-18658

Barriers and Facilitators to Healthcare Access among Sub-Saharan African Migrants in Europe: A Narrative Scoping Review

PLOS One

Dear Dr. Cynthia,

Thank you for submitting your manuscript to PLOS ONE. After careful consideration, we feel that it has merit but does not fully meet PLOS ONE’s publication criteria as it currently stands. Therefore, we invite you to submit a revised version of the manuscript that addresses the points raised during the review process.

Please submit your revised manuscript by Jun 20 2026 11:59PM. If you will need more time than this to complete your revisions, please reply to this message or contact the journal office at plosone@plos.org. When you're ready to submit your revision, log on to https://www.editorialmanager.com/pone/ and select the 'Submissions Needing Revision' folder to locate your manuscript file.

Please include the following items when submitting your revised manuscript:

- A letter that responds to each point raised by the academic editor and reviewer(s). You should upload this letter as a separate file labeled 'Response to Reviewers'.
- A marked-up copy of your manuscript that highlights changes made to the original version. You should upload this as a separate file labeled 'Revised Manuscript with Track Changes'.
- An unmarked version of your revised paper without tracked changes. You should upload this as a separate file labeled 'Manuscript'.

If you would like to make changes to your financial disclosure, please include your updated statement in your cover letter. Guidelines for resubmitting your figure files are available below the reviewer comments at the end of this letter.

If applicable, we recommend that you deposit your laboratory protocols in protocols.io to enhance the reproducibility of your results. Protocols.io assigns your protocol its own identifier (DOI) so that it can be cited independently in the future. For instructions see: https://journals.plos.org/plosone/s/submission-guidelines#loc-laboratory-protocols. Additionally, PLOS ONE offers an option for publishing peer-reviewed Lab Protocol articles, which describe protocols hosted on protocols.io. Read more information on sharing protocols at https://plos.org/protocols?utm_medium=editorial-email&utm_source=authorletters&utm_campaign=protocols.

As the corresponding author, your ORCID iD is verified in the submission system and will appear in the published article. PLOS supports the use of ORCID, and we encourage all coauthors to register for an ORCID iD and use it as well. Please encourage your coauthors to verify their ORCID iD within the submission system before final acceptance, as unverified ORCID iDs will not appear in the published article. *Only* the individual author can complete the verification step; PLOS staff *cannot* verify ORCID iDs on behalf of authors.

We look forward to receiving your revised manuscript.

Kind regards,

Felix Bongomin, MB ChB, Ph.D., FECMM

Academic Editor

PLOS One

**Journal Requirements:**

When submitting your revision, we need you to address these additional requirements.

1. Please ensure that your manuscript meets PLOS ONE's style requirements, including those for file naming. The PLOS ONE style templates can be found at

https://journals.plos.org/plosone/s/file?id=wjVg/PLOSOne_formatting_sample_main_body.pdf and

https://journals.plos.org/plosone/s/file?id=ba62/PLOSOne_formatting_sample_title_authors_affiliations.pdf

2. Please upload a new copy of Figures 1 and 2 as the detail is not clear. Please follow the link for more information:  https://journals.plos.org/plosone/s/figures

3. Please include captions for your Supporting Information files at the end of your manuscript, and update any in-text citations to match accordingly. Please see our Supporting Information guidelines for more information: http://journals.plos.org/plosone/s/supporting-information.

4. If the reviewer comments include a recommendation to cite specific previously published works, please review and evaluate these publications to determine whether they are relevant and should be cited. There is no requirement to cite these works unless the editor has indicated otherwise.

[Note: HTML markup is below. Please do not edit.]

Reviewers' comments:

Reviewer's Responses to Questions

**Comments to the Author**

1. Is the manuscript technically sound, and do the data support the conclusions?

The manuscript must describe a technically sound piece of scientific research with data that supports the conclusions. Experiments must have been conducted rigorously, with appropriate controls, replication, and sample sizes. The conclusions must be drawn appropriately based on the data presented.

Reviewer #1: Partly

**********

2. Has the statistical analysis been performed appropriately and rigorously?

Reviewer #1: N/A

**********

3. Have the authors made all data underlying the findings in their manuscript fully available?

The PLOS Data policy requires authors to make all data underlying the findings described in their manuscript fully available without restriction, with rare exception (please refer to the Data Availability Statement in the manuscript PDF file). The data should be provided as part of the manuscript or its supporting information, or deposited to a public repository. For example, in addition to summary statistics, the data points behind means, medians and variance measures should be available. If there are restrictions on publicly sharing data—e.g. participant privacy or use of data from a third party—those must be specified.

Reviewer #1: Yes

**********

4. Is the manuscript presented in an intelligible fashion and written in standard English?

PLOS ONE does not copyedit accepted manuscripts, so the language in submitted articles must be clear, correct, and unambiguous. Any typographical or grammatical errors should be corrected at revision, so please note any specific errors here.

Reviewer #1: No

**********

5. Review Comments to the Author

Please use the space provided to explain your answers to the questions above. You may also include additional comments for the author, including concerns about dual publication, research ethics, or publication ethics. (Please upload your review as an attachment if it exceeds 20,000 characters)

Reviewer #1: This manuscript addresses an important and timely topic—healthcare access among Sub-Saharan African (SSA) migrants in Europe—with clear policy and equity relevance. The focus on structural, cultural, and intersectional determinants is valuable, and the synthesis provides useful insights for research and practice.

However, there are significant methodological and reporting concerns that limit the rigor and clarity of the review. In particular, inconsistencies in review type, lack of a clearly defined review question, and insufficient transparency in data synthesis and methodological procedures need to be addressed before the manuscript can be considered for publication.

Major Comments

1. Conceptual and Methodological Inconsistency: The manuscript is described as a “narrative scoping review”, which is methodologically inconsistent. A review should be clearly defined as either a narrative review, or a scoping review with systematic methodology.

This issue appears throughout the manuscript (including title and abstract) . The authors should clearly define the review type and ensure consistency across all sections.

2. Although the authors state that they follow Arksey and O’Malley’s framework, a clear review question is not explicitly presented, which is a fundamental requirement of scoping reviews. The authors should

clearly articulate the primary review question, and demonstrate its alignment with the study objectives and eligibility criteria.

3-Use of Outdated Methodological Framework:The study relies primarily on Arksey and O’Malley (2005), with reference to Levac et al. . While foundational, this approach is now considered outdated if used alone. The manuscript would be strengthened by aligning with Joanna Briggs Institute (JBI) scoping review methodology, and/or clearly demonstrating how updated methodological refinements were applied.

4. The authors report using thematic synthesis for qualitative and mixed-methods evidence . However, the review includes quantitative studies (e.g., cross-sectional surveys and registry data). It is unclear how quantitative data were incorporated into the synthesis and whether a convergent or segregated approach was used. This requires clarification to ensure methodological transparency.

5-The inclusion criteria specify SSA migrants, but it is unclear how studies with mixed migrant populations were handled.

The authors should clarify whether such studies were included and how SSA-specific findings were extracted or interpreted.

6-The search strategy restricted Google Scholar screening to the first 10 pages .This approach requires justification and

supporting methodological references. Otherwise, it may introduce selection bias.

7- There is no mention of protocol registration (e.g., PROSPERO, OSF).Indicate whether a protocol exists, or justify its absence.

Minor Comments

1. The manuscript requires substantial language editing. Issues include:

Grammatical errors

Incorrect article usage

Long, complex sentences

For example, the Introduction is presented as a single extended paragraph, which reduces clarity and readability . It should be restructured into coherent sections.

Some sections (particularly Methods and Results) would benefit from More concise phrasing and clearer transitions between ideas.

2-Providing more detail on data extraction procedures and how to solve conflectes between revieweres.

3-The restriction to English-language studies only should be explicitly acknowledged as a limitation due to potential language bias.

**********

6. PLOS authors have the option to publish the peer review history of their article (what does this mean?). If published, this will include your full peer review and any attached files.

If you choose “no”, your identity will remain anonymous but your review may still be made public.

**Do you want your identity to be public for this peer review?** For information about this choice, including consent withdrawal, please see our Privacy Policy.

Reviewer #1: No

**********

[NOTE: If reviewer comments were submitted as an attachment file, they will be attached to this email and accessible via the submission site. Please log into your account, locate the manuscript record, and check for the action link "View Attachments". If this link does not appear, there are no attachment files.]

To ensure your figures meet our technical requirements, please review our figure guidelines: https://journals.plos.org/plosone/s/figures

You may also use PLOS’s free figure tool, NAAS, to help you prepare publication quality figures: https://journals.plos.org/plosone/s/figures#loc-tools-for-figure-preparation.

NAAS will assess whether your figures meet our technical requirements by comparing each figure against our figure specifications.

# Author comment: Author response to Decision Letter 1

**DOI:** 10.1371/journal.pone.0351011.r002

11 May 2026

Point-by-Point Response to Reviewers

We sincerely thank the reviewer for their thorough, constructive, and expert evaluation of our manuscript. The feedback has substantially improved the methodological rigour, clarity, and transparency of the review. Below, we address each comment point by point. All changes in the revised manuscript are highlighted in red to facilitate tracking. New references added in response to reviewers' comments are numbered 26, 27, and 29 and are highlighted in red in both the text and the reference list.

REVIEWER #1, MAJOR COMMENTS

Major Comment 1:

Conceptual and Methodological Inconsistency. Reviewer comment: "The manuscript is described as a 'narrative scoping review', which is methodologically inconsistent. A review should be clearly defined as either a narrative review, or a scoping review with systematic methodology. This issue appears throughout the manuscript (including title and abstract)."

Authors' response:

We fully accept this critique. The term 'narrative scoping review' was internally contradictory and has been removed throughout the manuscript. The review is now consistently described as a 'scoping review' in the:

• Title (changed from "A Narrative Scoping Review" to "A Scoping Review")

• Abstract Methods section

• Introduction (objectives paragraph)

• Methods, Study Design section

The revised Methods section now clearly situates the review as a scoping review following the Arksey and O'Malley framework, Levac et al. refinements, and the Joanna Briggs Institute (JBI) scoping review methodology.

Major Comment 2:

Absence of an Explicit Review Question. Reviewer comment: "Although the authors state that they follow Arksey and O'Malley's framework, a clear review question is not explicitly presented, which is a fundamental requirement of scoping reviews. The authors should clearly articulate the primary review question, and demonstrate its alignment with the study objectives and eligibility criteria."

Authors' response:

We agree that the absence of an explicit review question was a significant methodological gap. We have now added a clearly stated primary review question in both the Abstract and the Methods Study Design section, as follows:

"What are the barriers and facilitators to healthcare access and utilization among Sub-Saharan African migrants in Europe?"

The alignment between this review question, the study objectives, and the eligibility criteria is now explicitly stated in the revised Methods section. The inclusion criteria (SSA migrants; European setting; healthcare access/utilization focus; any study design; peer-reviewed) directly operationalize the boundaries implied by the review question. The three specific objectives map onto the scope defined by the question.

Major Comment 3:

Use of Outdated Methodological Framework. Reviewer comment: "The study relies primarily on Arksey and O'Malley (2005), with reference to Levac et al. While foundational, this approach is now considered outdated if used alone. The manuscript would be strengthened by aligning with Joanna Briggs Institute (JBI) scoping review methodology."

Authors' response:

We thank the reviewer for this important methodological feedback. We have added explicit reference to and alignment with the JBI scoping review methodology (Peters et al., 2020) [Reference 26], which represents the most current and comprehensive guidance for scoping review conduct. The revised Methods, Study Design section now reads as follows;

"In accordance with the Joanna Briggs Institute (JBI) scoping review methodology, the review was guided by a clearly defined primary review question... The review is explicitly defined as a scoping review, not a narrative review, in keeping with the systematic and iterative methodology employed throughout."

New reference added: Peters MDJ, Godfrey C, McInerney P, Munn Z, Tricco AC, Khalil H. Chapter 11: Scoping reviews (2020 version). In: Aromataris E, Munn Z, editors. JBI Manual for Evidence Synthesis. JBI; 2020. [Reference 26]

Major Comment 4:

Quantitative Data Integration in Thematic Synthesis. Reviewer comment: "The authors report using thematic synthesis for qualitative and mixed-methods evidence. However, the review includes quantitative studies (e.g., cross-sectional surveys and registry data). It is unclear how quantitative data were incorporated into the synthesis and whether a convergent or segregated approach was used."

Authors' response:

This is a valid and important methodological concern. We have substantially revised the Data Synthesis and Analysis section to clarify how quantitative data were incorporated. Specifically, we adopted a convergent integrated approach, consistent with Hong et al. (2017) [Reference 29]. In this approach:

• Quantitative findings were transformed into qualitative representations by extracting key outcomes, patterns, and statistically significant associations and recasting them as descriptive statements (e.g., registry evidence of lower utilisation among uninsured migrants was coded as "insurance as a structural barrier").

• These transformed quantitative contributions were then integrated alongside qualitative and mixed-methods findings into the thematic synthesis, enabling convergent interpretive themes to emerge across evidence types.

New reference added: Hong QN, Pluye P, Bujold M, Wassef M. Convergent and sequential synthesis designs: implications for conducting and reporting systematic reviews of qualitative and quantitative evidence. Syst Rev. 2017; 6(1):6. [Reference 29]

Major Comment 5:

Handling of Mixed Migrant Population Studies. Reviewer comment: "The inclusion criteria specify SSA migrants, but it is unclear how studies with mixed migrant populations were handled. The authors should clarify whether such studies were included and how SSA-specific findings were extracted or interpreted."

Authors' response:

We agree that clarification was necessary. We have added a dedicated paragraph following the Inclusion Criteria in the revised manuscript that reads as follows:

"Regarding studies with mixed migrant populations: studies that included both SSA migrants and migrants from other regions were included if they reported SSA-specific findings separately, or if the majority of the study sample comprised SSA migrants and disaggregated data were extractable. Where SSA-specific data could not be isolated from broader migrant population findings, the study was excluded to preserve analytical precision."

This clarification ensures transparency regarding how population specificity was maintained throughout the review.

Major Comment 6:

Justification for Google Scholar Search Restriction to First 10 Pages. Reviewer comment: "The search strategy restricted Google Scholar screening to the first 10 pages. This approach requires justification and supporting methodological references. Otherwise, it may introduce selection bias."

Authors' response:

We appreciate this comment. We have added a methodological justification and supporting reference for this decision in the revised Information Sources and Search Strategy section.

"This approach is consistent with established methodological guidance recommending that Google Scholar screening be restricted to the first 10 pages (200 results) in evidence reviews, given that beyond this threshold, results exhibit rapidly diminishing relevance to the search query. Notwithstanding, we acknowledge that this restriction may have resulted in missed records and should be considered a potential source of selection bias."

New reference added: Haddaway NR, Collins AM, Coughlin D, Kirk S. The role of Google Scholar in evidence reviews and its applicability to grey literature searching. PLoS One. 2015;10(9):e0138237. [Reference 27]

Major Comment 7:

Protocol Registration. Reviewer comment: "There is no mention of protocol registration (e.g., PROSPERO, OSF). Indicate whether a protocol exists, or justify its absence."

Authors' response:

We acknowledge this important point. We have added a clear statement on protocol registration in both the Methods, Study Design section and the Limitations section. The revised Methods text states:

"No formal protocol was registered with PROSPERO or the Open Science Framework prior to the conduct of this review. This decision reflects the exploratory and mapping purpose of the review, which did not meet PROSPERO's eligibility criteria for prospective systematic review registration. We acknowledge this as a methodological limitation and recommend that future scoping reviews on this topic consider prospective protocol registration to enhance transparency and reproducibility."

Additionally, the Limitations section now includes: "Fifth, no formal protocol was pre-registered for this review (e.g., on PROSPERO or the Open Science Framework), which limits the transparency and replicability of the review process. Future reviews on this topic are encouraged to register their protocol prospectively."

REVIEWER #1, MINOR COMMENTS

Minor Comment 1:

Language Editing and Introduction Structure. Reviewer comment: "The manuscript requires substantial language editing. Issues include grammatical errors, incorrect article usage, and long, complex sentences. The Introduction is presented as a single extended paragraph, which reduces clarity and readability."

Authors' response:

We have undertaken careful language revision throughout the manuscript. Specifically:

• The Introduction has been restructured from a single extended paragraph into four logically organized paragraphs:

(1) Migration context and SSA population profile in Europe (refs. 1–5)

(2) Structural, legal, and cultural barriers to healthcare (refs. 6–14)

(3) Intersectional vulnerabilities and life-course implications (refs. 15–20)

(4) Research gap and review objectives (refs. 21–22)

• Grammatical errors, incorrect article usage, and overly complex sentence constructions have been corrected throughout the manuscript.

• Transitions between ideas in the Methods and Results sections have been made more concise and coherent.

Minor Comment 2:

Detail on Data Extraction and Conflict Resolution. Reviewer comment: "Providing more detail on data extraction procedures and how to solve conflicts between reviewers."

Authors' response:

We have expanded the Data Charting Process subsection to provide more detail on both the data extraction procedures and the conflict resolution process. The revised text now clarifies that:

• The data extraction form was piloted on two studies before full-scale extraction, with refinements made as needed.

• When conflicts arose, primary reviewers attempted resolution through structured discussion; if unresolved after two cycles, a third independent reviewer (EAF or ANC) made the final adjudication.

• All resolved conflicts and the rationale for adjudication decisions were documented in the extraction log to ensure auditability.

Minor Comment 3:

English-Language Restriction as an Explicit Limitation. Reviewer comment: "The restriction to English-language studies only should be explicitly acknowledged as a limitation due to potential language bias."

Authors' response:

We agree. Although the English-language restriction was mentioned in the original Limitations section, it was insufficiently elaborated. We have strengthened this limitation statement in the revised manuscript as follows:

"Fourth, the restriction of the search to English-language publications constitutes a potential language bias: studies published in French, Dutch, German, Italian, Spanish, and other European languages were not retrieved, which may have led to the exclusion of relevant evidence from non-Anglophone European settings where SSA migrant communities are well-established (e.g., France, Portugal, Italy). This should be considered an inherent limitation of the evidence base."

We believe these revisions have substantially strengthened the manuscript. We are grateful for the reviewer's expert input and remain available to address any further concerns.

Attachment Submitted filename: Response to Reviewers.pdf

# Peer review: Decision Letter 1

**DOI:** 10.1371/journal.pone.0351011.r003

**Authors:** Felix Bongomin

22 May 2026

Barriers and Facilitators to Healthcare Access among Sub-Saharan African Migrants in Europe: A  Scoping Review

PONE-D-26-18658R1

Dear Dr. Cynthia,

We’re pleased to inform you that your manuscript has been judged scientifically suitable for publication and will be formally accepted for publication once it meets all outstanding technical requirements.

Within one week, you’ll receive an e-mail detailing the required amendments. When these have been addressed, you’ll receive a formal acceptance letter and your manuscript will be scheduled for publication.

An invoice will be generated when your article is formally accepted. Please note, if your institution has a publishing partnership with PLOS and your article meets the relevant criteria, all or part of your publication costs will be covered. Please make sure your user information is up-to-date by logging into Editorial Manager at Editorial Manager® and clicking the ‘Update My Information' link at the top of the page. For questions related to billing, please contact billing support.

If your institution or institutions have a press office, please notify them about your upcoming paper to help maximize its impact. If they’ll be preparing press materials, please inform our press team as soon as possible -- no later than 48 hours after receiving the formal acceptance. Your manuscript will remain under strict press embargo until 2 pm Eastern Time on the date of publication. For more information, please contact onepress@plos.org.

Kind regards,

Felix Bongomin, MB ChB, Ph.D., FECMM

Academic Editor

PLOS One

Additional Editor Comments (optional):

Reviewers' comments:

Reviewer's Responses to Questions

**Comments to the Author**

1. If the authors have adequately addressed your comments raised in a previous round of review and you feel that this manuscript is now acceptable for publication, you may indicate that here to bypass the “Comments to the Author” section, enter your conflict of interest statement in the “Confidential to Editor” section, and submit your "Accept" recommendation.

Reviewer #1: All comments have been addressed

**********

2. Is the manuscript technically sound, and do the data support the conclusions?

The manuscript must describe a technically sound piece of scientific research with data that supports the conclusions. Experiments must have been conducted rigorously, with appropriate controls, replication, and sample sizes. The conclusions must be drawn appropriately based on the data presented.

Reviewer #1: Yes

**********

3. Has the statistical analysis been performed appropriately and rigorously?

Reviewer #1: N/A

**********

4. Have the authors made all data underlying the findings in their manuscript fully available?

The PLOS Data policy requires authors to make all data underlying the findings described in their manuscript fully available without restriction, with rare exception (please refer to the Data Availability Statement in the manuscript PDF file). The data should be provided as part of the manuscript or its supporting information, or deposited to a public repository. For example, in addition to summary statistics, the data points behind means, medians and variance measures should be available. If there are restrictions on publicly sharing data—e.g. participant privacy or use of data from a third party—those must be specified.

Reviewer #1: Yes

**********

5. Is the manuscript presented in an intelligible fashion and written in standard English?

PLOS ONE does not copyedit accepted manuscripts, so the language in submitted articles must be clear, correct, and unambiguous. Any typographical or grammatical errors should be corrected at revision, so please note any specific errors here.

Reviewer #1: (No Response)

**********

6. Review Comments to the Author

Please use the space provided to explain your answers to the questions above. You may also include additional comments for the author, including concerns about dual publication, research ethics, or publication ethics. (Please upload your review as an attachment if it exceeds 20,000 characters)

Reviewer #1: Thank you for addressing my comments. In future, I suggest to use JBI methodology and follow it step by step.

**********

7. PLOS authors have the option to publish the peer review history of their article (what does this mean?). If published, this will include your full peer review and any attached files.

If you choose “no”, your identity will remain anonymous but your review may still be made public.

**Do you want your identity to be public for this peer review?** For information about this choice, including consent withdrawal, please see our Privacy Policy.

Reviewer #1: No

**********

# Editor report: Acceptance letter

**DOI:** 10.1371/journal.pone.0351011.r004

**Authors:** Felix Bongomin

PONE-D-26-18658R1

PLOS One

Dear Dr. Cynthia,

I'm pleased to inform you that your manuscript has been deemed suitable for publication in PLOS One. Congratulations! Your manuscript is now being handed over to our production team.

At this stage, our production department will prepare your paper for publication. This includes ensuring the following:

* All references, tables, and figures are properly cited

* All relevant supporting information is included in the manuscript submission,

* There are no issues that prevent the paper from being properly typeset

You will receive further instructions from the production team, including instructions on how to review your proof when it is ready. Please keep in mind that we are working through a large volume of accepted articles, so please give us a few days to review your paper and let you know the next and final steps.

Lastly, if your institution or institutions have a press office, please let them know about your upcoming paper now to help maximize its impact. If they'll be preparing press materials, please inform our press team within the next 48 hours. Your manuscript will remain under strict press embargo until 2 pm Eastern Time on the date of publication. For more information, please contact onepress@plos.org.

You will receive an invoice from PLOS for your publication fee after your manuscript has reached the completed accept phase. If you receive an email requesting payment before acceptance or for any other service, this may be a phishing scheme. Learn how to identify phishing emails and protect your accounts at https://explore.plos.org/phishing.

If we can help with anything else, please email us at customercare@plos.org.

Thank you for submitting your work to PLOS ONE and supporting open access.

Kind regards,

PLOS ONE Editorial Office Staff

on behalf of

Dr. Felix Bongomin

Academic Editor

PLOS One
